# Generation and Functional Characterization of Anti-CD19 Chimeric Antigen Receptor-Natural Killer Cells from Human Induced Pluripotent Stem Cells

**DOI:** 10.3390/ijms241310508

**Published:** 2023-06-22

**Authors:** Phatchanat Klaihmon, Xing Kang, Surapol Issaragrisil, Sudjit Luanpitpong

**Affiliations:** 1 Siriraj Center of Excellence for Stem Cell Research, Faculty of Medicine Siriraj Hospital, Mahidol University, Bangkok 10700, Thailand; p.klaihmon@gmail.com (P.K.); xing.kang001@gmail.com (X.K.); surapolsi@gmail.com (S.I.); 2 Division of Hematology, Department of Medicine, Faculty of Medicine Siriraj Hospital, Mahidol University, Bangkok 10700, Thailand; 3 BDMS Center of Excellence for Hematology, Wattanosoth Cancer Hospital, Bangkok 10310, Thailand; 4 Blood Products and Cellular Immunotherapy Research Group, Faculty of Medicine Siriraj Hospital, Mahidol University, Bangkok 10700, Thailand

**Keywords:** chimeric antigen receptor, induced pluripotent stem cell, natural killer cell, anti-CD19 CAR, leukemia, CAR-NK, NK differentiation

## Abstract

Natural killer (NK) cells are a part of innate immunity that can be activated rapidly in response to malignant transformed cells without prior sensitization. Engineering NK cells to express chimeric antigen receptors (CARs) allows them to be directed against corresponding target tumor antigens. CAR-NK cells are regarded as a promising candidate for cellular immunotherapy alternatives to conventional CAR-T cells, due to the relatively low risk of graft-versus-host disease and safer clinical profile. Human induced pluripotent stem cells (iPSCs) are a promising renewable cell source of clinical NK cells. In the present study, we successfully introduced a third-generation CAR targeting CD19, which was validated to have effective signaling domains suitable for NK cells, into umbilical cord blood NK-derived iPSCs, followed by a single-cell clone selection and thorough iPSC characterization. The established single-cell clone of CAR19-NK/iPSCs, which is highly desirable for clinical application, can be differentiated using serum- and feeder-free protocols into functional CAR19-iNK-like cells with improved anti-tumor activity against CD19-positive hematologic cancer cells when compared with wild-type (WT)-iNK-like cells. With the feasibility of being an alternative source for off-the-shelf CAR-NK cells, a library of single-cell clones of CAR-engineered NK/iPSCs targeting different tumor antigens may be created for future clinical application.

## 1. Introduction

Cancer has been the second leading cause of death globally for many years and is attributable to one in six deaths. By 2040, the global burden of cancer is projected to be 28.4 million new cases, a 47% rise from 2020 [1]. The mainstays of cancer treatment have been surgery, chemotherapy, and radiation, and, more recently, targeted therapies using monoclonal antibodies and small molecule inhibitors [2,3]. Although these approaches have contributed to improved outcomes, certain cancers still carry a poor prognosis. Immune escape is recognized as an emerging hallmark of cancers, leading to the concept of cancer immunotherapy to restore immunity to keep cancer permanently at bay [2]. Adoptive cellular immunotherapy employing engineered T cells expressing chimeric antigen receptor (CAR), which is designed to mimic T-cell receptor (TCR) signaling, has demonstrated promising anti-tumor effects. Although various CAR-T-cell platforms targeting CD19 have demonstrated impressive remission rates for the treatment of relapsed/refractory acute lymphoblastic leukemia (ALL), diffuse large B-cell lymphoma (DLBCL), and mantle cell lymphoma (MCL), for example [4], there are a few outstanding challenges for the engineering of autologous CAR-T cells, which limit its general use [5]. Due to the laborious and time-consuming process, autologous CAR-T cells are not suitable for patients with rapidly progressing disease and patients with a limited number of functional T cells.

Natural killer (NK) cells are part of the first line of defense in the innate immunity, with a potent ability to kill malignant transformed cells and tumor cells without prior sensitization. NK cell-mediated cytotoxicity depends on the balance between signals from activating receptors, e.g., CD16, natural cytotoxicity receptor family members (NKp30, NKp40, NKp44, and NKp46), killer immunoglobulin-like receptors (KIRs), and NKG2D, and inhibitory receptors [6,7]. Trials using allogeneic NK cells from various sources, including peripheral blood, umbilical cord blood (UCB), and the NK-92 cell line, have demonstrated that NK cells are well tolerated in patients without requiring human leukocyte antigen (HLA) matching, with little evidence of graft-versus-host disease (GvHD) [8,9], eliminating the need to produce personalized, autologous CAR-NK cells for each patient. Additionally, the administration of CAR-NK cells was not associated with the development of cytokine release syndrome (CRS) and neurotoxicity in phase 1 and 2 clinical trials [10].

The breakthrough in human induced pluripotent stem cell (iPSC) research has opened up vast opportunities for regenerative medicine. iPSCs are an ideal starting material for next-generation CAR-related immune cell therapy, due to the ease of genetic manipulation and limitless expansion during the iPSC stage [11,12]. Currently, several phase 1 clinical trials have utilized allogeneic iPSCs as the source of NK cells for treatment of various solid tumors and hematologic malignancies, with or without engineered CAR [13,14]. Previously, we designed a third-generation CAR targeting CD19 that employs CD28, 4-1BB, and CD3ζ, similar to the framework of CAR-T cells, and showed that such a construct was feasible to generate CAR-NK-92 cells with high and selective cytotoxicity against CD19-positive tumor cells [15]. In the present study, we investigated the feasibility of generating third-generation CAR-NK cells targeting CD19 from iPSCs using serum- and feeder-free protocols, which may be further established into Good Manufacturing Practice (GMP)-compliant protocols. The iPSC line MUSIi013-A, which is a single-cell clone of iPSCs established from NK cells collected from human UCB (NK/iPSC) [16], was chosen as a starting cell source because it may contain epigenetic memory that predisposes iPSCs to enhanced NK differentiation [17,18]. Upon introduction of anti-CD19 CAR into NK/iPSCs, we performed single-cell clone selection and comprehensive iPSC characterization. We hypothesized that the differentiated anti-CD19 CAR-expressing NK cells would have improved anti-tumor activity and amplified NK receptor signaling. Our study outlines the initial concept and production platform for generation of CAR-NK cells from a single-cell clone of CAR-NK/iPSCs, which may be useful for the generation of other single-cell clones of iPSC lines and subsequent NK or other immune cells expressing CARs. 

## 2. Results

### 2.1. Engineering the Anti-CD19 CAR-Expressing NK/iPSC Line

**Figure 1:**
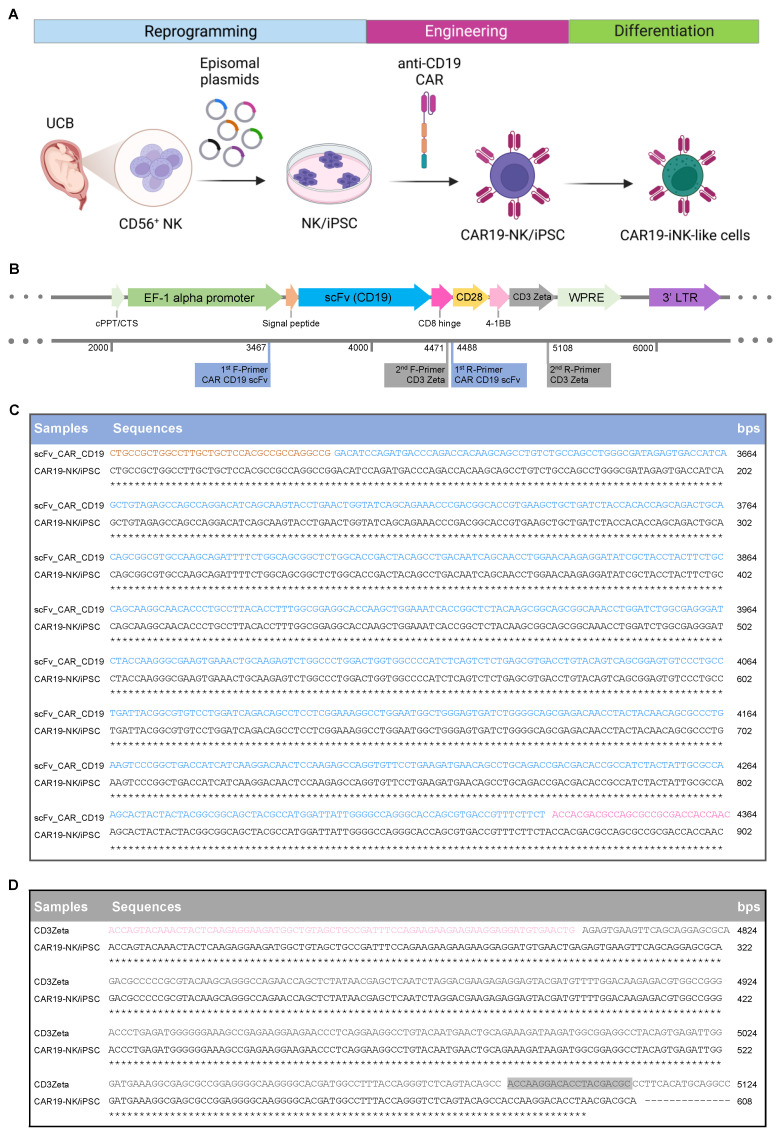
Construction of anti-CD19 CAR and its introduction into iPSCs. (**A**) Schematic overview outlining UCB-derived NK cell reprogramming, CAR engineering to establish single-cell clones of CAR19-NK/iPSCs, and their subsequent differentiation into NK cells, referred to as CAR19-iNK-like cells. (**B**) (**Upper**) Diagram of the third-generation anti-CD19 CAR construct harboring signal peptide, anti-CD19 scFv fragment, CD8 hinge, CD28, 4-1BB, and CD3ζ under the control of EF-1 alpha promoter. (**Lower**) Two sets of primers used for DNA sequencing and their predicted target regions are shown. (**C**,**D**) DNA sequencing results of CAR19-NK/iPSCs (clone 5-19H10) spanning over the scFv fragment (**C**) and CD3ζ (**D**). Color coding denotes the regions of CAR construct—orange denotes signal peptide, blue denotes scFv fragment, pink denotes CD8 hinge, light pink denotes 4-1BB, and grey denotes CD3ζ. Grey shading illustrates primer target. Asterisk (*) denotes identical sequence between CAR construct and CAR19-NK/iPSCs.

**Figure 2:**
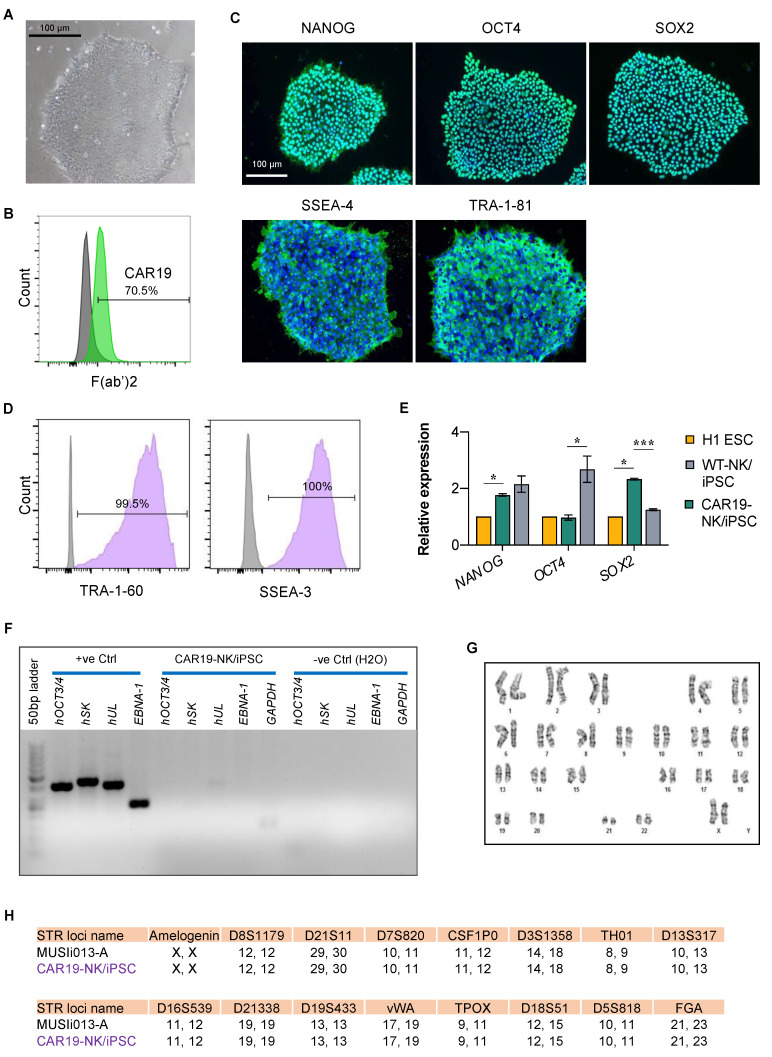
Characterization of CAR19-NK/iPSCs. (**A**) Representative micrograph of CAR19-NK/iPSC colony. (**B**) CAR expression on the surface of CAR19-NK/iPSCs as evaluated by flow cytometry using an anti-F(ab′)2 antibody. Data are shown as a histogram overlay: isotype control (grey) and CAR19-NK/iPSCs (green). (**C**) Representative micrographs of immunofluorescence staining showing the pluripotency markers NANOG, OCT4, SOX2, SSEA-4, and TRA-1-81. (**D**) Quantitative analysis of TRA-1-60- and SSEA-3-positive cells. Data are shown as a histogram overlay: isotype control (grey) and CAR19-NK/iPSCs (purple) (**E**) mRNA expression of *NANOG*, *OCT4*, and *SOX2* by qPCR. Data are mean ± SEM (*n* = 3). * *p* < 0.05, *** *p* < 0.001 versus H1 ESC or WT-NK/iPSCs; unpaired *t*-test. (**F**) Evidence of the loss of episomal transgenes detected by PCR. (**G**) Karyotype analysis as determined by G-banding assay. (**H**) STR analysis comparing a total of 16 loci between the parental MUSIi013-A and CAR19-NK/iPSC lines.

Previously, we generated an NK/iPSC line from CD3^−^CD56^+^CD16^+^ NK cells isolated from UCB from a healthy newborn by using non-integrable episomal reprogramming [16]. Three episomal plasmids encoding SOX2, KLF4, L-MYC, LIN28, OCT3/4, and shRNA against *TP53* and an extra plasmid encoding transient EBNA-1, which enhanced the reprogramming efficiency [19], were introduced to NK cells by 4D nucleofection. Following the morphology-based clonal selection, thorough iPSC characterization and validation were performed and the established NK/iPSC line was designated and registered as MUSIi013-A. To provide an alternative source of CAR-expressing NK cells other than immortalized NK-92 cells, which have to be irradiated before infusion into patients [20], we transduced MUSIi013-A NK/iPSCs with the third-generation anti-CD19 CAR using lentiviral particles, as schematically depicted in Figure 1A. Our CAR design and construct, which harbors an anti-CD19 scFv fragment consisting of heavy and light chains linked to human CD28, 4-1BB, and CD3ζ signaling domains via a CD8 hinge region with a signal peptide sequence (MALPVTALLLPLALLLHAARP) (Figure 1B), were successfully used to generate efficient anti-CD19 CAR-NK-92 cells in the previous study [15]. After transduction, selected iPSC clones emerging from a single cell were picked in the presence of small-molecule cocktail of four inhibitors (SMC4), consisting of SB431542 (TGFβi), PD0325901 (MEKi), CHIR99021(GSKi), and Thiazovivin (ROCK) [21], and screened for scFv expression on the cell surface via flow cytometry using an anti-F(ab′)2 antibody. The overall efficiency of single-cell clone selection and CAR integration was 4.17% (4/96). Clone 5-19H10 was picked and genomic DNA sequencing was performed to confirm the insertion of anti-CD19 CAR using two sets of target primers. Nucleotide sequences of the selected clone were aligned to the sequences of the CAR cassette at the scFv and CD8 hinge, and 4-1BB and CD3ζ regions, which revealed 100% identity, excluding the poor base calling at the start of the trace (Figure 1C,D). The CAR expression of the selected clone, hereafter called CAR19-NK/iPSC line (Figure 2A), was confirmed once again by flow cytometry using an anti-F(ab′)2 fragment antibody (Figure 2B).

### 2.2. Characterization of CAR19-NK/iPSCs and Its Multilineage Differentiation

**Figure 3:**
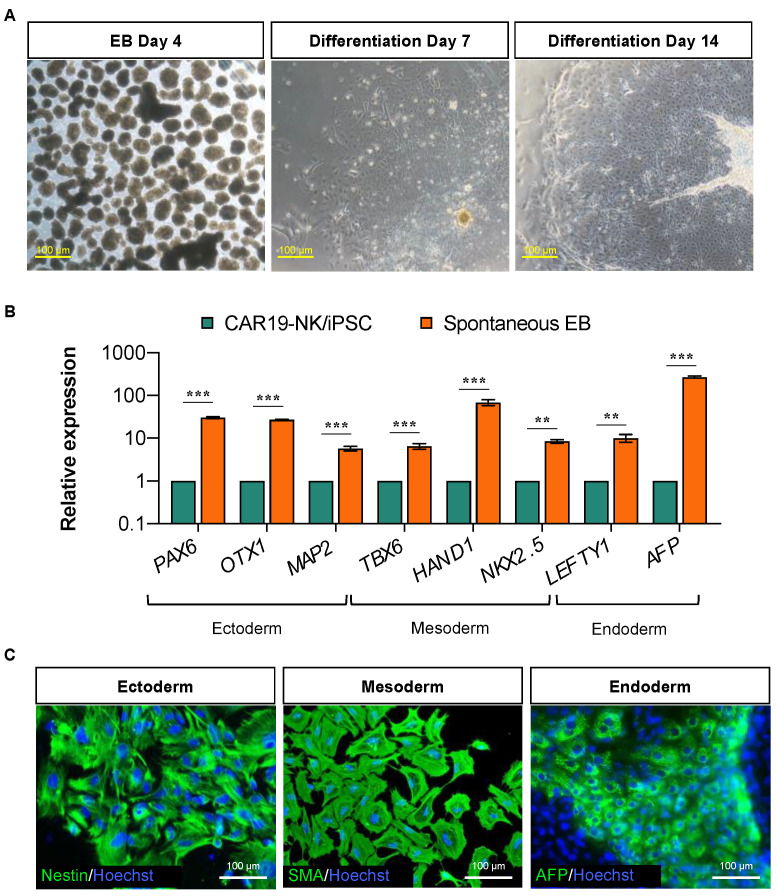
Differentiation potential of CAR19-NK/iPSCs as evaluated by EB formation. (**A**) Morphology of floating EBs on days 4, 7, and 14 of differentiation. (**B**) mRNA expression of trilineage differentiation markers: *PAX6*, *OTX1*, and *MAP2* for ectoderm; *TBX6*, *HAND1*, and *NKX2.5* for mesoderm; and *LEFTY1* and *AFP* for endoderm. Data are mean ± SD (*n* = 3). ** *p* < 0.01, *** *p* < 0.001 versus undifferentiated CAR19-NK/iPSCs; unpaired *t*-test. (**C**) Representative micrographs of immunofluorescence staining of trilineage differentiation markers.

We next validated that CAR19-NK/iPSCs retained its pluripotency by determining the presence of well-characterized and widely accepted pluripotency markers using immunofluorescence (NANOG, OCT4, SOX2, SSEA-4, and TRA-1-81) (Figure 2C), flow cytometry (TRA-1-60 and SSEA-3) (Figure 2D), and quantitative real-time PCR (qPCR; *OCT4*, *NANOG*, and *SOX2*) in comparison to H1 ESC and wild-type (WT)-NK/iPSCs (Figure 2E). The loss of episomal transgenes in CAR19-NK/iPSCs was confirmed once again by PCR (Figure 2F). Transduction of CAR did not induce aneuploidy in CAR19-NK/iPSCs as determined by karyotype analysis at passage 28, displaying normal karyotype (46XX) at 400–500 resolution (Figure 2G). Short tandem repeat (STR) analysis confirmed that CAR19-NK/iPSCs shared a perfect match with their parental NK/iPSCs and UCB-derived NK cells (Figure 2H). For a functional assay on the ability of the cells to differentiate into ectoderm, endoderm, and mesoderm, we utilized spontaneous in vitro differentiation by embryoid body (EB) formation. The morphology of floating EBs on day 4 was compared with the morphology of the same EBs on days 7 and 14 of differentiation (Figure 3A). Figure 3B shows a remarkable upregulation of the representative markers for ectoderm (*PAX6*, *OTX1*, and *MAP2*), mesoderm (*TBX6*, *HAND1*, and *NKX2.5*), and endoderm (*LEFTY1* and *AFP*) in EBs on day 14 of differentiation when compared to non-differentiated cells. Immunofluorescence showing the expression of markers for endoderm (alpha fetoprotein, AFP), mesoderm (smooth muscle actin, SMA), and ectoderm (Nestin) further confirmed the in vitro differentiation (Figure 3C).

### 2.3. Induction of Hematopoietic Progenitor Cells from CAR19-NK/iPSCs

**Figure 4:**
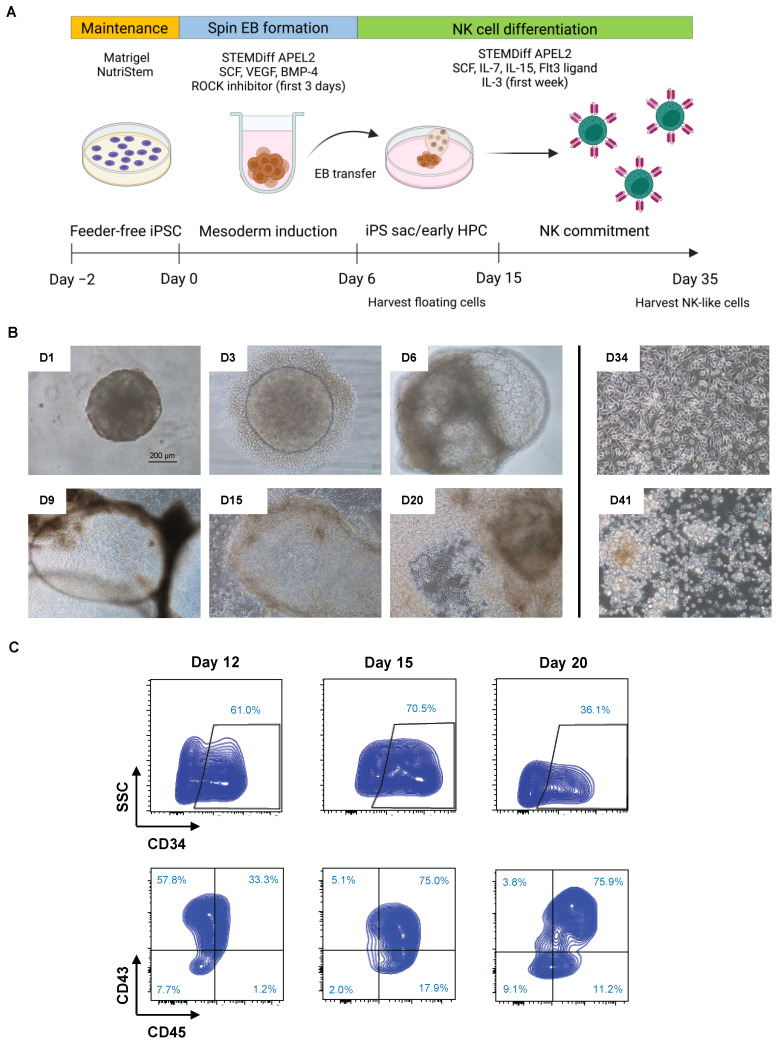
Generation of spin EBs and induction of HPCs from CAR19-NK/iPSCs. (**A**) Schematic diagram showing a two-stage protocol for the differentiation of NK cells from iPSCs under serum- and feeder-free conditions. (**B**) Morphology of generated EBs on various days of culture. iPS sacs were observed on day 9 onwards and completely disappeared on day 34. (**C**) Representative flow cytometric analysis of the HPC markers CD34, CD43, and CD45 on days 12, 15, and 20 of culture.

We aimed to produce functional anti-CD19 CAR-NK cells from NK/iPSCs using serum- and feeder-free protocols devoid of animal-derived products. We adopted the differentiation protocols used by Lupo et al. [22], which consisted of two major steps—the mesoderm induction to generate hematopoietic progenitor cells (HPCs) from iPSCs using spin EB formation and the differentiation of CD34^+^ HPCs into NK cells, with slight modifications, as schematically depicted in Figure 4A. Optimizations for spin EB formation were first carried out with CAR19-NK/iPSCs. Briefly, CAR19-NK/iPSCs were spin-aggregated in an ultra-low attachment, round-bottom, 96-well plate at a density of 5000 cells/well, which was higher than the original protocol at a density of 3000 cells/well. The morphology of floating EBs on days 1, 3, and 6 after mesoderm induction was considered healthy, as they maintained a round morphology and exhibited continued growth (Figure 4B, upper). Upon modification of the cell seeding density, generated EBs were transferred onto the Matrigel-coated plate as early as day 6 (originally on day 11) after spin EB formation and cultured in NK cell differentiation medium afterwards. On day 9 of culture, inflated sac-like structures (iPS sacs), which contained round hematopoietic-like cells, appeared (Figure 4B, middle). Next, floating cells were collected from the iPS sacs and analyzed for the HPC markers CD34, CD43, and CD45 using flow cytometry. Figure 4C shows that the majority of floating cells on days 12 and 15 were CD34^+^, mostly with CD43^+^, thus confirming that they were HPCs. We observed a decrease in CD34^+^ floating cells on day 20 onwards, indicating that HPCs started to differentiate into more mature cells in the NK lineage. On days 34 and 41 of culture, the iPS sacs completely disappeared, while free-floating, multicellular aggregates were observed (Figure 4B, lower).

### 2.4. CAR19-NK/iPSC-Derived HPCs Can Be Differentiated toward the NK-Cell Lineage

**Figure 5:**
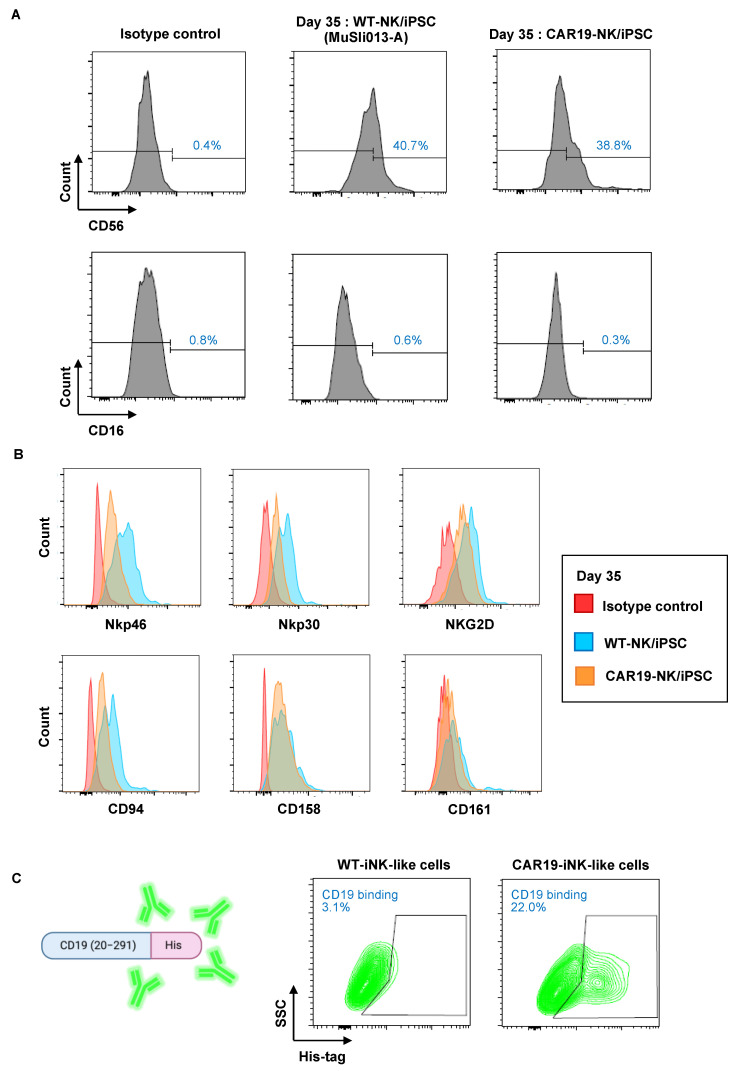
Immunophenotypic profiles of iNK-like cells derived from CAR19- and WT-NK/iPSCs. (**A**) Flow cytometric analysis of typical NK surface markers CD56 and CD16 on day 35 of culture of CAR19-NK/iPSCs and parental WT-NK/iPSCs. (**B**) Flow cytometric analysis of NK lineage commitment CD161, activating receptors NKG2D, Nkp46, and Nkp30, KIRs CD158, and CD94. (**C**) Anti-CD19 CAR expression (green, box) in the differentiated CAR19-iNK-like cells as evaluated by flow cytometry based on its binding activity to the specific antigen His tag-rhCD19.

Next, CAR19-NK/iPSCs and WT-NK/iPSCs were differentiated using the optimized spin EB formation and transfer. Free-floating, multicellular aggregates from CAR19-NK/iPSCs and WT-NK/iPSCs were collected on day 35 and analyzed for the typical human NK cell surface markers CD56 and CD16 by flow cytometry. Approximately 40% of the differentiated cells from CAR19-NK/iPSCs and WT-NK/iPSCs were CD56^+^CD16^−^ cells (Figure 5A), similar to the main subset of NK cells found in the lymph nodes. Further, the differentiated cells were phenotypically characterized using common NK cell activation markers, which were grouped into: (i) a marker of NK lineage commitment CD161; (ii) activating receptors NKG2D, Nkp46, and Nkp30; (iii) KIRs CD158; and (iv) CD94, which is normally associated with NKG2A to form receptors for MHC class I molecules [6,7,23]. Figure 5B shows that the differentiated cells from both CAR19-NK/iPSCs and WT-NK/iPSCs expressed all tested NK cell activation markers, indicating that CAR19-NK/iPSCs and WT-NK/iPSCs were capable of differentiation into NK cell lineage and NK-like cells via EB-derived HPCs. The binding activity of NK-like cells derived from CAR19-NK/iPSCs, hereafter called CAR19-iNK-like cells, to recombinant human CD19 (20–291) antigen fused with a His tag confirmed that they retained anti-CD19 CAR expression (Figure 5C).

### 2.5. Selective Cytotoxicity of CAR19-NK/iPSC-Derived NK-like Cells against CD19-Positive Hematologic Cancer Cells

**Figure 6:**
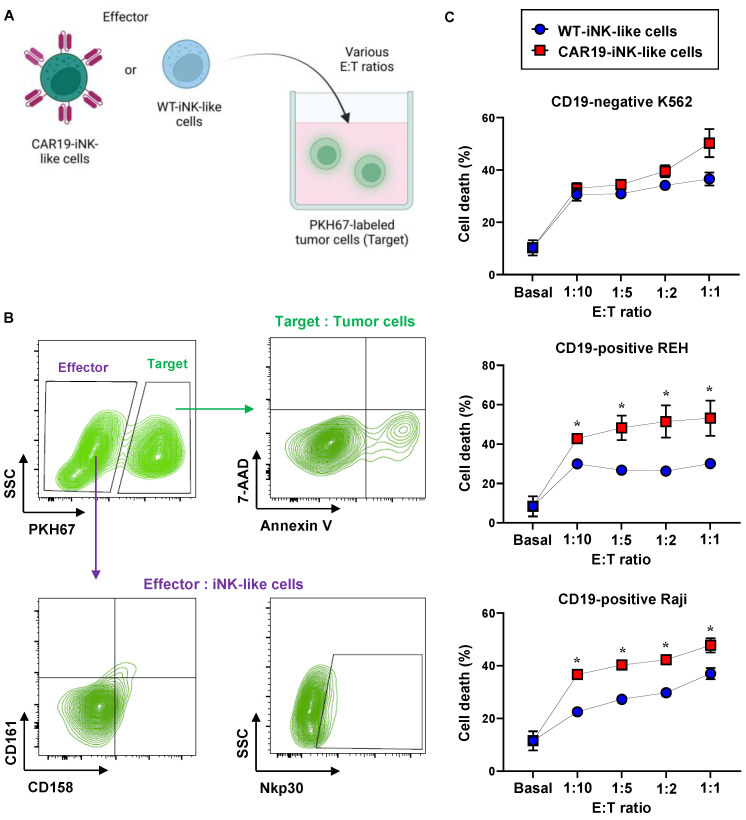
CAR19-iNK-like cells selectively induced apoptosis of CD19-positive target tumor cells. (**A**) Schematic diagram of the NK cell cytotoxicity assay showing the labeling of target tumor cells with PKH67 before exposure to effector cells at various E:T ratios. (**B**) Flow cytometry gating strategy to specifically detect cell death in PKH67-labeled target tumor cells and NK cell markers in effector cells. (**C**) Percentages of total tumor cell death, comprising Annexin V- and/or 7-AAD-positive cells, in CD19-negative K562 cells and CD19-positive REH and Raji cells at the indicated E:T ratio were plotted. Data are mean ± SEM (*n* = 3). * *p* < 0.05 versus WT-iNK-like cells under the same conditions; unpaired *t*-test.

The cytotoxicity of CAR19-iNK-like cells was tested against CD19-positive target tumor cells, including human acute lymphoblastic leukemia (ALL)-derived REH cells and human Burkitt’s lymphoma (BL)-derived Raji cells. REH and Raji cells, so-called target cells (T), were labeled with PKH67 green fluorescence and cocultured with CAR19-iNK-like or WT-NK/iPSC-derived NK-like (WT-iNK-like) cells, so-called effector cells (E), at E:T ratios of 1:10, 1:5, 1:2, and 1:1 for 4 h, as schematically depicted in Figure 6A. PKH67-positive target cells were then evaluated for cell death by Annexin V/7-AAD assay using the gating strategy shown in Figure 6B. Human chronic myeloid leukemia (CML)-derived K562 cells, which are CD19-negative cells, were also used herein to confirm the specificity of cytotoxicity of CAR19-iNK-like cells. Figure 6C shows that CAR19-iNK-like exerted superior anti-tumor activity against REH and Raji cells when compared with WT-iNK-like cells, while having a minimal effect on K562 cells, indicating that they were functionally competent with high specificity toward target tumor cells presenting CD19 antigen.

### 2.6. Activation of NK Cell Receptors in CAR19-NK/iPSC-Derived NK-like Cells upon Tumor Exposure

**Figure 7:**
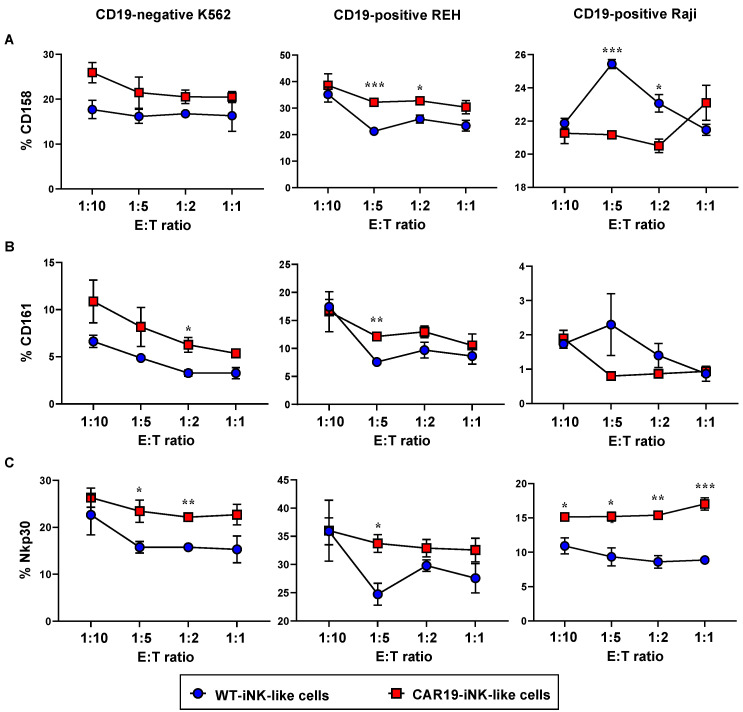
Expression of NK cell activation markers in CAR19- and WT-iNK-like cells upon exposure to hematologic cancer cells. Surface CD158 (**A**), CD161 (**B**), and Nkp30 (**C**) in CAR19- and WT-iNK-like cells in response to K562, REH, and Raji cells at various E:T ratios were evaluated by flow cytometry, and their percentages were plotted. Data are mean ± SEM (*n* = 3). * *p* < 0.05, ** *p* < 0.01, and *** *p* < 0.001 versus WT-iNK-like cells under the same conditions; unpaired *t*-test.

To confirm that the anti-tumor activity of CAR19-iNK-like cells was mediated via NK signaling, we measured NK cell activation markers, including CD161, CD158, and Nkp30, upon exposure of the cells to PKH67-labeled target tumor cells using the gating strategy shown in Figure 6B. Figure 7A–C shows that exposure of NK-like cells to different tumor cells yielded different activation patterns. For example, while CAR19-iNK-like cells significantly upregulated NKp30 in response to all tested tumor cells when compared with WT-iNK-like cells, particularly at the low E:T ratios, they activated CD158 in REH cells and inhibited CD158 in Raji cells. Notably, the activation of Nkp30 by CAR19-iNK-like cells in CD19-negative K562 cells suggests the CAR-independent cytotoxicity. Altogether, our data strengthen the theory that the differentiated cells, either CAR19-iNK-like or WT-iNK-like cells, exerted NK phenotypes.

## 3. Discussion

NK cell-based immunotherapy, including that of CAR-NK cells, has been used in several clinical trials, due to its potent ability to kill tumor cells with a low risk of major complications such as GvHD, acute CRS, and neurotoxicity. Herein, we were able to introduce the third-generation CAR, targeting CD19, which was validated to have effective signaling domains suitable for NK cells, in human NK-derived iPSCs to subsequently generate anti-CD19 CAR-NK-like cells, referred to as CAR19-iNK-like cells.

CAR-NK cells have constituted a promising area of cellular immunotherapy innovation, particularly allogeneic CAR-NK cells, which are recognized as a promising off-the-shelf product. The majority of ongoing clinical trials involve CAR-NK-92 cells, which modified the FDA-approved NK-92 cell line, for treatment of both hematologic cancers and solid tumors [7,24]. Earlier, we demonstrated the feasibility of designing third-generation CARs and generating CAR-NK-92 cells targeting CD19, CD138, or other tumor antigens, which support the idea of generating a library of CAR-NK cells targeting different antigens for the personalized proof of concept [15]. However, the major drawback of NK-92-based cellular immunotherapy is the malignant origin of parental NK-92 cells—they were derived from a patient with non-Hodgkin’s lymphoma with severe chronic Epstein–Barr virus (EBV) infection [25,26]. Hence, CAR-NK-92 cells need to be irradiated before clinical application to avoid the secondary tumorigenesis and potential EBV susceptibility. After irradiation, CAR-NK-92 cells cannot proliferate, and the survival time in vivo is relatively short, resulting in a limited persistence. Peripheral blood (PB)-derived NK cells are another important source of clinical NK cells. The proportion of PB-NK cells is less than 15% of all PB lymphocytes, hence they need to be activated and expanded before the introduction of CARs [27], as the obtained cells are likely limited to a few individual doses.

Recently, iPSCs have represented an attractive cell source for producing a large number of NK cells due to the limitless expansion during the iPSC stage [13,28,29]. Primary NK cells generally have relatively low transduction efficiency compared to T cells, due to resistance to viral transduction from innate defense mechanisms guided by pattern recognition receptors recognizing foreign genetic material [30]. The use of iPSCs for CAR-NK cell production would be advantageous due to the ease of genetic manipulation. Indeed, we found that anti-CD19 CAR can be introduced to NK/iPSCs and that a single-cell clone of CAR19-NK/iPSCs, which is highly desirable for clinical application, can be established in the presence of SMC4 (Figure 1) and that CAR19-iNK-like cells derived from CAR19-NK/iPSCs retained anti-CD19 CAR expression and selective cytotoxicity towards CD19-positive tumor cells (Figure 5 and Figure 6). The differentiated WT-iNK-like cells from WT-NK/iPSCs, even without anti-CD19 CAR, demonstrated an anti-tumor activity against certain tested tumor cells, likely because NK cells may target tumor cells in an antigen-unrestricted manner [31,32]. Characterization of the differentiated CAR19- and WT-iNK-like cells revealed that they were negative for CD16 (Figure 5), consistent with previous studies showing immature, low CD16 in iPSC-derived NK cells [29,33]; therefore, tumor cells could not be eliminated through CD16-mediated antibody-dependent cell-mediated cytotoxicity (ADCC). The anti-tumor activity might be improved by introducing a CD16 transgene, similar to the FT596—the first iPSC-derived CAR-NK cell product that underwent a clinical trial (NCT04245722)—which expressed a high-affinity, non-cleavable CD16 with an IL-15 receptor fusion protein (IL-15/R-hnCD16) [24]. With the optimized CAR design and established protocols for the generation of a single-cell clone of CAR-engineered NK/iPSCs, a library of CAR-engineered NK/iPSCs targeting a wide variety of tumor antigens could be created to be applied as an alternative cell source for off-the-shelf allogeneic CAR-NK products for future clinical application.

It is important to note that we may further improve NK cell differentiation by using different approaches to improve the differentiation efficiency at the stage of HPC differentiation via mesoderm induction and/or NK commitment. Previous studies have demonstrated that bone morphogenetic protein 4 (BMP4), fibroblast growth factor 2 (FGF2), WNT/β-catenin, and Activin/Nodal play critical roles for mesoderm formation [34,35,36,37]. Given that BMP4, SCF, and VEGF have already been supplemented in the used hematopoietic differentiation medium, we may additionally include growth factors or small molecule inhibitors that manipulate WNT/β-catenin and Activin/Nodal signaling, e.g., Activin A, CHIR99021 (GSK-3 inhibitor), and SB-431542 (TGFβ inhibitor) [14]. Moreover, we are currently investigating the roles of *O*-GlcNAcylation, a nutrient-sensitive posttranslational modification of proteins, in HPC differentiation from iPSCs, as we previously found that *O*-GlcNAcylation is a key determinant of hematopoietic stem cell (HSC) fate decision and certain lineage-specific differentiation processes, including megakaryopoiesis [38], erythropoiesis [39], and dendritic cell differentiation [40]. For improving NK commitment, we may further increase the concentrations of major cytokines, such as IL-7 and IL-15, or introduce an additional stage of lymphoid progenitor expansion before NK cell commitment.

## 4. Materials and Methods

### 4.1. Cell Culture

Human iPSCs, MUSIi013-A single-cell clone, reprogrammed from UCB-derived NK cells (NK/iPSC) [15] were routinely cultivated in NutriStem XF medium (Sartorius, Göttingen, Germany) on Matrigel-coated plates (Corning, Corning, New York, NY, USA) and mechanically passaged every two to three days. HEK293FT cells were cultured in high-glucose DMEM (DMEM-HG) medium while hematologic cancer cell lines, including K562, REH, and Raji cells (American Type Culture Collection, Manassas, VA, USA) were cultured in RPMI 1640 medium. DMEM-HG and RPMI1640 medium were supplemented with 10% FBS, L-glutamine, and antibiotics (Gibco, Thermo Fisher Scientific, Waltham, MA, USA). All cells were culture at 37 °C and 5% CO_2_.

### 4.2. Lentiviral Particle Production

Lentiviral production was performed using HEK293FT packaging cells in conjunction with pCMV.dR8.2 dvpr lentiviral packaging and pCMV-VSV-G envelope plasmids (Addgene #8454 and #8455) [41]. Briefly, HEK293FT cells were transfected with anti-CD19 CAR (Creative Biolabs, Shirley, New York, NY, USA), pCMV.dR8.2 dvpr, and pCMV-VSV-G plasmids at the ratio of 12:5:1 using Lipofectamine 3000 (Thermo Fisher Scientific, Waltham, MA, USA). The lentiviral particles were harvested and pooled at approximately 48 h post-transfection and were concentrated using Amicon Ultra-15 centrifugal filters (Merck Millipore, Tullagreen, Ireland).

### 4.3. CAR Transduction and Single-Cell Clone Isolation

MUSIi013-A cells were transduced with lentiviral particles carrying the anti-CD19 in the presence of 4 μg/mL hexadimethrine bromide (Sigma-Aldrich, St. Louis, MO, USA). Single cells were seeded on irradiated human foreskin fibroblasts in NutriStem medium supplemented with SMC4 small-molecule cocktail inhibitors containing PD0325901 (Sigma Aldrich, St. Louis, MO, USA), CHIR99021, Thiazovivin, and SB431542 (STEMCELL Technologies, Vancouver, BC, Canada) [21]. Emerging single-cell colonies were manually picked up and cultured on Matrigel-coated plates and underwent full iPSC characterization. Clone 5-19H10 (CAR19-NK/iPSC) and its parental cells (WT-NK/iPSC) were used in this study.

### 4.4. Genomic DNA Sequencing

Genomic DNA was isolated using a PureLink Genomic DNA Mini Kit (Invitrogen, Waltham, MA, USA). The target regions for DNA sequencing were amplified by PCR using Q5 High-Fidelity DNA Polymerase (New England Biolabs, Ipswich, MA, USA) with specific primers, and the resulting PCR products were purified by a GenepHlow Gel/PCR kit (Geneaid, New Taipei City, Taiwan). A total of 0.2 μg PCR product was then used for DNA sequencing using ABI PRISM BigDye^TM^ Terminator Cycle Sequencing Kit v3.1 (1st BASE, Singapore).

### 4.5. Evaluation of CAR Expression

CAR expression in iPSCs was determined by flow cytometry based on Fab fragments. Cells were incubated with FITC-conjugated anti-mouse-IgG, F(ab′)2 fragment antibody (F(ab′)2-FITC; Jackson ImmunoResearch, West Grove, PA, USA) for 30 min at 4 °C and analyzed using a BD FACS Canto (BD Biosciences, San Jose, CA, USA).

CAR expression in the differentiated cells was evaluated by target antigen-based detection to ascertain its binding activity. Briefly, cells were incubated with 10 μg/mL rhCD19 (20–291) protein with His tag to the C-terminus (Abcam, Cambridge, UK) for 1 h at 4 °C, followed by an incubation with FITC-conjugated anti-His tag antibody (His tag-FITC; Abcam) for 15 min at room temperature and flow cytometric analysis. Cells that were incubated with His tag-FITC, but not with rhCD19, were used as a basal control. The percentage of cells that expressed CAR could be calculated from the subtraction of FITC-positive cells in the basal control from those with the target protein.

### 4.6. Immunofluorescence Staining

Cells were fixed in 4% paraformaldehyde for 20 min, permeabilized with 0.1% Triton X-100/PBS for 10 min, and blocked with 3% BSA/PBS for 1 h. The cells were incubated with primary antibodies in 1% BSA/PBS overnight at 4 °C, followed by secondary antibodies at room temperature for 1 h. Nuclei were counterstained with Hoechst 33342 (Thermo Fisher Scientific) for 30 min at room temperature and visualized under fluorescence microscope (Eclipse Ti-U, Nikon, Tokyo, Japan) with NIS-Elements D Software (version 4.30.00; Nikon).

### 4.7. Flow Cytometry

For analysis of pluripotency markers, iPSCs were dissociated into single cells using TrypLE^TM^ Select (Gibco), blocked with 10% human AB serum, stained with Alexa Fluor 488-SSEA-3 and Alexa Fluor 647-TRA-1-60 (BioLegend, San Diego, CA, USA), and analyzed by BD FACS Canto flow cytometer (BD Biosciences) with FlowJo software (V10.4.1). For phenotypic analysis of HPC induction and NK commitment, the cells were resuspended in FACS buffer (PBS with 2% bovine serum albumin), incubated with antibody cocktail for 30 min at 4 °C in the dark, and analyzed by BD FACS Canto flow cytometer. The antibodies used in this study included FITC-CD16, FITC-Nkp46, PE-CD56, PE-Nkp44, PerCP-CD45, PerCP/Cy5.5-CD94, APC-CD34, PE/Cy7-CD43, PE/Cy7-KIR, Alexa Fluor 700-CD161, and BV605-Nkp30 (BioLegend).

### 4.8. qPCR Analysis

Total RNA was isolated using TRI Reagent^®^ (Molecular Research Center, Cincinnati, Ohio, USA) and converted to complementary DNA using the RevertAid First Strand cDNA synthesis kit (Thermo Fisher Scientific). The qPCR reactions were performed on the CFX384 Touch Real-Time PCR detection system (Bio-Rad, Hercules, CA, USA) using SYBR^TM^ Select Master Mix (Thermo Fisher Scientific). The cycle parameters started with an activation step at 95 °C for 2 min, followed by 40 cycles of denaturation at 95 °C for 15 s and annealing/extension at 60 °C for 1 min.

### 4.9. Karyotyping

The standard G-banded karyotyping was performed at the Department of Obstetrics and Gynecology, Faculty of Medicine Siriraj Hospital, Mahidol University. A total of 25 metaphases at a band resolution of 400–450 were analyzed.

### 4.10. STR Analysis

STR analysis was performed at the Department of Forensic Medicine, Faculty of Medicine Siriraj Hospital, Mahidol University. A total of 16 loci were tested.

### 4.11. Spontaneous In Vitro Differentiation via EB Formation

The iPSCs were harvested into small clumps using 1 mg/mL Dispase (Gibco) and cultured on low attachment dishes in knockout DMEM supplemented with 20% knockout serum replacement, 2 mM GlutaMAX^TM^, 0.1 mM MEM non-essential amino acid, 0.1 mM β-mercaptoethanol, 1× insulin-transferrin-selenium-ethanolamine, and 100 U/mL penicillin/streptomycin (Gibco). The medium was replaced every other day. On day 7, EBs were transferred onto a 0.1% gelatin-coated plate and cultured at 37 °C and 5% CO_2_ for another 3 weeks.

### 4.12. NK Cell Differentiation

iPSCs were pre-treated with ROCK inhibitor for 1 h before single cell dissociation by Accutase^TM^ (Gibco). The HPC induction protocol was slightly modified from the protocol used by Lupo KB, et al. [22] by means of cell density and duration of each phase. Briefly, 5000 single cells were seeded in each well of an ultra-low attachment, round-bottom 96-well plate in 100 μL hematopoietic differentiation medium comprising STEMdiff APEL2 medium (STEMCELL Technologies), 40 ng/mL stem cell factor (SCF), 20 ng/mL bone morphogenic protein-4 (BMP-4), 20 ng/mL vascular endothelial growth factor (VEGF), and 10 μM ROCK inhibitor for the first 3 days. All cytokines used were obtained from R&D Systems (Minneapolis, MN, USA). The plate was centrifuged at 250× *g* for 5 min to promote EB formation and incubated for 6 days, during which the medium was changed every 3 days by removing 70 μL of medium from each well and adding 100 μL of freshly prepared medium without ROCK inhibitor.

On day 6, 30 EBs were transferred onto a Matrigel-coated 6-well plate in 4 mL NK cell differentiation medium consisting of STEMdiff APEL2 medium, 20 ng/mL SCF, 20 ng/mL IL-7, 10 ng/mL IL-15, and 10 ng/mL Flt3 ligand, which was supplemented with 5 ng/mL IL-3 for the first week. The medium was half-changed every two to three days for 3–4 weeks.

### 4.13. NK Cell Cytotoxicity Assay

Target tumor cells were labeled with PKH67 lipophilic dye for 5 min at 37 °C and were subsequently incubated with the corresponding ratio of effector CAR19- and WT-iNK-like cells in a total concentration of 20,000 cells per well for 4 h at 37 °C in a round-bottom 96-well plate. After that, the cell mixture was harvested and stained with PE-conjugated annexin-V and 7-AAD (BD Biosciences, Franklin Lakes, NJ, USA) in Ca^2+^-rich buffer for 15 min at room temperature. Samples were analyzed immediately via FACS analysis. Total cell death of target tumor cells was defined as annexin-V and/or 7-AAD-positive cells in the PKH67-positive cell population.

### 4.14. Statistical Analysis

Prism 9 (GraphPad software, San Diego, CA, USA) was used for all data and statistical analysis, with a *p*-value < 0.05 considered to be significant. Data represent mean ± SD or mean ± SEM from three or more independent experiments as indicated. Statistical analysis between two groups was performed by Student’s unpaired *t*-test at a significance level of *p* < 0.05.

## 5. Conclusions

In summary, our findings reveal a promising CAR design and production protocol for the establishment of a single-cell clone of CAR-engineered NK/iPSCs, which is highly desirable for clinical application. We also showed its feasibility to be differentiated into CAR-iNK-like cells, which displayed high and selective cytotoxicity toward corresponding leukemia and lymphoma cells in vitro, using serum- and feeder-free protocols. Further studies should focus on improving differentiation efficiency at the stages of HPC induction and/or NK commitment, as well as an expansion of differentiated CAR-iNK-like cells to obtain a sufficient number of cells for clinical applications.

## Data Availability

The data used to support the findings of this study are included within the article.
